# Higenamine Attenuates Neuropathic Pain by Inhibition of NOX2/ROS/TRP/P38 Mitogen-Activated Protein Kinase/NF-ĸB Signaling Pathway

**DOI:** 10.3389/fphar.2021.716684

**Published:** 2021-09-24

**Authors:** Bing Yang, Shengsuo Ma, Chunlan Zhang, Jianxin Sun, Di Zhang, Shiquan Chang, Yi Lin, Guoping Zhao

**Affiliations:** School of Traditional Chinese Medicine, Jinan University, Guangzhou, China

**Keywords:** higenamine, neuropathic pain, oxidative stress, neuroinflammation, transient receptor potential, NOX2

## Abstract

Oxidative stress damage is known as one of the important factors that induce neuropathic pain (NP). Using antioxidant therapy usually achieves an obvious curative effect and alleviates NP. Previous pharmacological studies have shown that higenamine (Hig) performs to be antioxidant and anti-inflammatory. However, the protective effect and mechanism of Hig on NP are still unclear. This study mainly evaluated the changes in reactive oxygen species (ROS) level, lipid peroxidation, and antioxidant system composed of superoxide dismutase (SOD) and glutathione (GSH) through chronic constrict injury (CCI) model rats and t-BHP-induced Schwann cell (SC) oxidative stress model. The expressions of two inflammatory factors, tumor necrosis factor-alpha (TNF-α) and interleukin-6 (IL-6), were also assessed. The possible molecular mechanism of Hig in the treatment of NP was explored in conjunction with the expression of mitochondrial apoptosis pathway and NOX2/ROS/TRP/P38 mitogen-activated protein kinase (MAPK)/NF-ĸB pathway-related indicators. Hig showed substantial antioxidant and anti-inflammatory properties both *in vivo* and *in vitro*. Hig significantly reduced the upregulated levels of ROS, malondialdehyde (MDA), TNF-α, and IL-6 and increased the levels of SOD and GSH, which rebalanced the redox system and improved the survival rate of cells. In the animal behavioral test, it was also observed that Hig relieved the CCI-induced pain, indicating that Hig had a pain relief effect. Our research results suggested that Hig improved NP-induced oxidative stress injury, inflammation, and apoptosis, and this neuroprotective effect may be related to the NOX2/ROS/TRP/P38 MAPK/NF-ĸB signaling pathway.

## Introduction

Neuropathic pain (NP) is a chronic pain condition caused by nervous system damage or disease leading to abnormal signals in the somatosensory system (Finnerup et al., 2021). NP is usually accompanied by hyperalgesia and/or allodynia manifested as burning and tingling sensations (Campbell and Meyer, 2006). At present, a variety of clinical diseases give rise to NP, mainly including metabolic or nutritional nerve changes, viral infections, and accompanying nerve damage. With the increasing incidence of metabolic diseases and the application of cancer chemotherapy, the rate of NP has risen year by year, which not only brings a great impact on patients but also seriously increases the social and economic burden (Colloca et al., 2017). It is widely accepted that the pathophysiology of evoked pain involves peripheral and central sensitization. Alterations in ion channels and interactions between cells and molecular signaling transmission are based on the sensitization of nociceptive pathways (Campbell and Meyer, 2006; Zhang et al., 2017). Previous researches showed that oxidative stress and inflammation are also important mechanisms in inducing and maintaining NP and they are in the pathological process of NP throughout (Costigan et al., 2009; Areti et al., 2014). The products of oxidative stress and inflammation, such as reactive oxygen species (ROS), tumor necrosis factor-α (TNF-α), and interleukin-6 (IL-6), promote each other *via* specific signaling pathways, aggravate the release of these factors, cause irreversible damage to cells, and even lead to cell apoptosis. The clinical treatment of NP includes drug therapy and interventional therapy, of which drug therapy is the main one. As the common treatment drugs, antidepressants and anticonvulsants (gabapentin and pregabalin, for example) have shown only partial pain relief effect, always followed by side effects to patients (Baron et al., 2010). Therefore, the development of new therapeutic drugs is still a hot topic at present.

Higenamine (Hig) is a plant-based alkaloid with antioxidation (Guler et al., 2020), anti-inflammatory (Yang et al., 2020a), and antiapoptosis (Yang et al., 2020b) effects. Hig is mainly used to scavenge oxygen free radicals to achieve antioxidant effects (Romeo et al., 2020). Our previous study showed that Hig inhibited the production of ROS in hypothermia-induced oxidative stress and also prevented the transport of α2C-AR from the cytoplasm to the membrane in hypothermic HDMECs, which indicated that Hig may reduce the cold-induced vasoconstriction (Guan et al., 2019). Besides, Hig is one of the tetrahydroisoquinolinic derivatives and there is a report speculating that it may be provided with neuroprotective effects (Peana et al., 2019). Indeed, existing studies have shown that Hig protected brain/reimplantation (I/R) damage by inhibiting Akt and Nrf2/HO-1 signaling pathways against oxygen-glucose deprivation/reperfusion-induced injury (Zhang et al., 2019). And Hig showed the neuroprotective effect on Alzheimer’s disease model rats by improving the cognitive impairment, regulating enzyme activity, and reducing cytotoxicity to inhibit oxidative stress damage (Yang et al., 2020b). Although more and more researches in recent year have proved that Hig has a good effect on many diseases, its effect and mechanism of how to act on NP are still unclear; thus, further research is needed by far.

This present study mainly explored the biological effects and potential molecular mechanisms of Hig on NP and demonstrated that Hig may regulate NP *via* NOX2/ROS/TRP/P38 mitogen-activated protein kinase (MAPK)/NF-ĸB signaling pathway, providing an experimental basis for the research and development of novel drugs.

## Materials and Methods

### Chemical Reagent

Demethylcoclaurine hydrochloride (≥98%) was purchased from Shanghai Yuanye Bio-Technology Co., Ltd. (Shanghai, China). Tert-butyl hydroperoxide (t-BHP) was from Energy Chemical (Shanghai, China). Dulbecco’s modified Eagle’s medium (DMEM), Penicillin-Streptomycin solution, and 0.25% trypsin were obtained from Gibco (Grand Island, NY, United States). Fetal bovine serum (FBS) was from Double-Helix (Beijing, China). Cell Counting Kit-8 (CCK-8) was purchase from Beijing Biosynthesis Biotechnology Co., Ltd. (Beijing, China). ROS assay kit, microreduced glutathione (GSH) assay kit, and mitochondrial membrane potential (MMP) assay kit with JC-1 were from Beijing Solarbio Science & Technology Co., Ltd. (Beijing, China). Annexin V-FITC/PI apoptosis kit was obtained from Shanghai Yishan Biotechnology Co., Ltd. (Shanghai, China). Hoechst 33258/PI Apoptosis Assay Kit was from Beyotime Biotechnology (Shanghai, China). Malondialdehyde (MDA) assay kit and superoxide dismutase (SOD) assay kit were from Nanjing Jiancheng Bioengineering Institute (Nanjing, Jiangsu, China). IL-6, IL-1β, TNF-α, and ROS ELISA kit were obtained from Wuhan Meimian Biotechnology Co., Ltd. (Wuhan, Hubei, China). RNAiso Plus (Trizol) was purchased from TaKaRa (Tokyo, Japan). Evo M-MLV RT Kit with gDNA Clean for qPCR and SYBR® Green Premix Pro Taq HS qPCR Kit were from Accurate Biology (Changsha, Hunan, China). FGSuper Sensitive ECL Luminescence Reagent was purchased from Meilunbio® (Dalian, Liaoning, China). All of the primary antibodies were obtained from Cell Signaling Technology unless otherwise stated (Danvers, MA, United States).

### Cell Culture

The immortal rat Schwann cell 96 (RSC96) was purchased from iCell Bioscience Inc. (Shanghai, China). RSC96 is cultivated in a complete medium made up of 90% DMEM, 10% FBS, and 1% Penicillin-Streptomycin solution in a sterile environment with 5% CO_2_ and 37°C.

### Cell Viability Assay

CCK-8 was used to evaluate cell viability. RSC96 was plated in a 96-well plate with a density of 5 × 10^3^ cells/well and treated with a different drug concentration. Then 100 µL of cell culture complete medium containing 10% CCK-8 solution was added to 96-well plate and incubated for 1 h at 37°C. The absorbance values (450 nm) were detected by a microplate reader (BioTek, United States).

### Cell Treatment

Hig was dissolved in dimethyl sulfoxide (DMSO). As previous research reported, t-BHP was performed to establish an oxidase stress injury model on cells (Zhao et al., 2017). RSC96 was pretreated with different concentration of Hig for 12 h and then t-BHP with a complete medium was added to each well for 2 h to assess the effect of Hig on t-BHP-exposed RSC96.

### Analysis of ROS

The intracellular ROS levels were measured by fluorometry and flow cytometry using 2,7-dichlorodi-hydrofluorescein diacetate (DCFH-DA) dye. The DCFH-DA probe was mixed with FBS-free medium at 1:2,000 (1:5,000 for flow cytometry). The volume of mixed medium to cell cultural plate was added appropriately to cover the cells and incubated for 20 min at 37°C. After that, RSC96 was washed by an FBS-free medium three times to completely remove fluorescent probes. The ROS level was observed under a fluorescence microscope (Carl Zeiss AG, Germany) and analyzed by flow cytometer (Beckman Coulter, Inc., United States).

### Determinations of GSH, SOD, and MDA

The intracellular levels of GSH, SOD, and MDA contents were measured according to the manufacturer’s instructions. In brief, RSC96 was washed and resuspended with PBS after administration. The same RSC96 volume of GSH extract was added three times to resuspend the cells, frozen and thawed twice in liquid nitrogen, and centrifuged to aspirate the supernatant for testing. Samples and reagents were mixed following the instructions, which were reacted at room temperature for 2 min, and the absorbance was measured at a wavelength of 412 nm. As for the extraction of SOD and MDA, ultrasonication was applied to gain cellular SOD and MDA. The absorbance of SOD and MDA was detected, respectively, at 550 and 532 nm with the microplate reader (BioTek, United States).

### Hoechst 33258/PI Staining

The Hoechst 33258/PI staining of RSC96 was performed to observe the state of cell apoptosis and necrosis directly. A suitable cover glass was put into the cell plate in advance and the glass was washed gently with PBS after administration. 95% ethanol was used to fix cell for 15 min at room temperature. Then, the surface liquid was dried, and 10 μL Hoechst 33258/PI solution was added on it and mounted. The changes of the apoptotic and necrotic nucleus were observed and photographed by fluorescence microscopy (Olympus, Japan).

### Flow Cytometry

Flow cytometry was used to account for the apoptosis rate of RSC96 and detect the functional condition of the mitochondrion, which were measured through Annexin V-FITC/PI staining and JC-1 staining, respectively. In brief, after administration, the cell culture medium was drawn into a prelabeled 15 ml centrifuge tube, RSC96 was washed twice with cold PBS, and PBS was drawn again into the corresponding centrifuge tube. 0.25% trypsin without EDTA digested cell for 1 min and was centrifuged at 1500 rpm for 5 min. RSC96 were resuspended in 500 µL 1× binding buffer, stained with 5 µL Annexin V-FITC and 10 µL PI solution, incubated at room temperature for 5 min in the dark environment, and immediately examined by flow cytometer (Beckman Coulter, Inc., United States). As for the detection of MMP, RSC96 were resuspended in 0.5 ml cell culture medium, mixed with 0.5 ml JC-1 staining solution, and incubated for 20 min at 37°C, 5% CO_2_. After the incubation, the cells were centrifuged at 4°C and 600 g for 3 min, washed with prechilled 1 × JC-1 staining buffer solution twice, and 1 ml 1 × JC-1 staining buffer solution was added to resuspend RSC96, followed by analyzing the condition of MMP with a flow cytometer (Beckman Coulter, Inc., United States).

### Real-Time Quantitative Polymerase Chain Reaction

RSC96 was washed with cold PBS after treatment. RNAiso Plus was applied to extract total RNA and the detective values of OD260/280 were used to quantify total RNA. Evo M-MLV RT Kit with gDNA Clean for qPCR was used to reverse transcription according to the manufacturer’s protocol. Amplification was performed in the CFX Connect Real-Time PCR Detection System (BIO-RAD, Hercules, CA, United States) following the protocol of SYBR® Green Premix Pro Taq HS qPCR Kit. The reaction procedure was as follows: one cycle at 95°C for 30 s for predenaturation and 40 cycles at 95°C for 5 s and at 60°C for 30 s for reaction. The primer GAPDH (B661204) was purchased from Sangon Biotech. Co., Ltd. (Shanghai, China) and the primers of TNF-α IL-6 and IL-1β were also designed by them which were listed in Table 1. The 2−ΔΔCT method was used to analyze expression levels of TNF-α IL-6 and IL-1β. Each sample was measured three times and averaged.

**TABLE 1:** Primer sequences for RT-qPCR.

Gene	Forward (5′-3′)	Reverse (5′-3′)
TNF-α	CGT​CGT​AGC​AAA​CCA​CCA​AG	CAC​AGA​GCA​ATG​ACT​CCA​AAG
IL-6	ACT​TCC​AGC​CAG​TTG​CCT​TCT​TG	TGG​TCT​GTT​GTG​GGT​GGT​ATC​CTC
IL-1β	CTC​ACA​GCA​GCA​TCT​CGA​CAA​GAG	TCC​ACG​GGC​AAG​ACA​TAG​GTA​GC

### Animal

Forty-eight specific pathogen-free (SPF) grade adult male Sprague Dawley (SD) rats were provided by Beijing Huafukang Biotechnology Co., Ltd. (Beijing, China, certificate number SCXK (Jing) 2019-0008). The rats were kept in a 12 h light/dark cycle environment with a temperature of 20–25°C and a humidity of 50–70%. Animal test procedures and general handling comply with the ethical guidelines and standards for the care and use of laboratory animals established by the Animal Test Ethics Committee of Jinan University. All efforts were made to minimize animal suffering.

### CCI Surgery

The rats were randomly divided into sham-operated group, CCI model group, and low/medium/high concentration of Hig and gabapentin (GBP) treatment group with eight rats in each group. The specific surgical procedures have been introduced in our previous study (Zhang et al., 2020).

### Pharmacological Treatment

The rats accepted medical treatment with oral administration after surgery. Rats in low/medium/high concentration of Hig and GBP group were treated with 25/50/100 mg·kg^−1^·d^−1^ Hig and 50 mg·kg^−1^·d^−1^ GBP, respectively. Those rats in the sham-operated and CCI groups were not treated with drugs but 6 ml·kg^−1^·d^−1^ 0.9% saline.

### Behavioral Tests

von Frey filaments (Ugo Basile Biological Research Apparatus, Varese, Italy) were applied to evaluate mechanical allodynia in CCI surgical rats while a hot-plate test was used to assess heat hyperalgesia. The von Frey test and hot-plate test were detailly described in our previous research (Zhang et al., 2020) and they were performed the day before the surgery and days 3, 5, 7, 10, 14, and 21 after surgery.

### Enzyme-Linked Immunosorbent Assay

Ipsilateral L4/5 dorsal root ganglion (DRG) was collected to detect the expression level of ROS while serum was collected for inflammatory factors (TNF-α, IL-6, and IL-1β). DRG was weighted and PBS was added to adjust its concentration to 0.1 g/ml. It was homogenized thoroughly and centrifuged at 4°C and 3000 rpm for 20 min. The supernatant was carefully aspirated for testing. And serum was diluted for detection. The experimental operation was performed according to the ELISA kits’ instructions and the absorbance was measured at 450 nm with a microplate reader (BioTek, United States).

### Measurement of GSH in DRGs

The fresh DRGs are washed twice with PBS and then 0.1 g of the tissue is weighted and homogenized. The remaining steps were carried out according to the kit manufacturer’s instructions.

### Histopathology

The right sciatic nerve tissues were fixed and washed with water, dehydrated, transparent, and embedded in paraffin. After the slices were processed in 5 µm thick sections, rehydrated, stained with hematoxylin-eosin (HE), washed with ethanol, dehydrated, and made transparent with xylene, they were observed and photographed under the microscope (Olympus, Japan).

### Western Blotting Analysis

The obtained RSC96 and ipsilateral L4/5 DRG tissue were used to perform the WB analysis. Cell and tissues were lysed with RIPA lysis buffer (Solarbio Science & Technology Co., Ltd, Beijing, China) and the protein concentration was quantified using BCA Protein Assay Kit (Solarbio Science & Technology Co., Ltd., Beijing, China). 10% SDS-PAGE was carried out to separate obtained protein samples and the proteins were transferred to polyvinylidene fluoride (PVDF) membrane (Millipore, MA, United States). 5% nonfat milk was used to block the membranes for 2 h at room temperature. Primary antibodies, including Bcl-2, Bax, caspase-3, cleaved caspase-3, cytochrome-c, p38 MAPK, phospho-p38 MAPK, phospho-NF-κB p65, GAPDH, TRPA1 (Novus Biologicals, United States), TRPV1 (Novus Biologicals, United States), and anti-NOX2/gp91phox (Abcam Corporation, England), were diluted in 1:1,000. Then, the membranes were put into primary antibodies above at 4°C for incubating overnight. After washing with TBST three times, the diluted secondary primary antibody was applied to incubate the membranes at 4°C for 2 h. TBST was used to wash the obtained protein bands and they were exposed using FGSuper Sensitive ECL Luminescence Reagent.

### Molecular Docking

The molecular structure of Hig was downloaded from PubChem database and the three-dimensional structure of Nox2, TRPA1, and TRPV1 protein from the RCSB Protein Data Bank. The AutodockTools 1.5.6 software was used to deal with these structures and determine the coordinates and box size of the Vina molecule docking. In order to increase the accuracy of the calculation, the parameter exhaustiveness was set to 20. Then Autodock Vina 1.1.2 software was performed to do semiflexible docking of molecules and proteins for selecting the best conformation of affinity. The docking binding mode was used to analyze the conformation with the lowest docking score and finally plot it in the Pymol software. Besides, the three-dimensional structures of Nox2 and TRPV1, Nox2, and TRPA1 protein were processed in pymol and protein docking was performed in ZDOCK 3.0.2. As a result, the top ten complexes were selected and scored and the best scores were chosen for mapping.

### Statistical Analysis

GraphPad Prism 8 and SPSS 22.0 were used for statistical analyses. All data were expressed as mean ± SEM. All experiments in this study were performed in triplicate. One-way ANOVA was used for comparison between groups, followed by Turkey tests. The behavioral data were analyzed using multivariate analysis of variance in the general linear model to compare data among the groups at each time point, followed by the Student–Newman–Keuls tests. *p* value less than 0.05 was considered to be statistically significant.

## Results

### Hig Showed a Protective Effect Against T-BHP-Induced Cell Cytotoxicity

**FIGURE 1:**
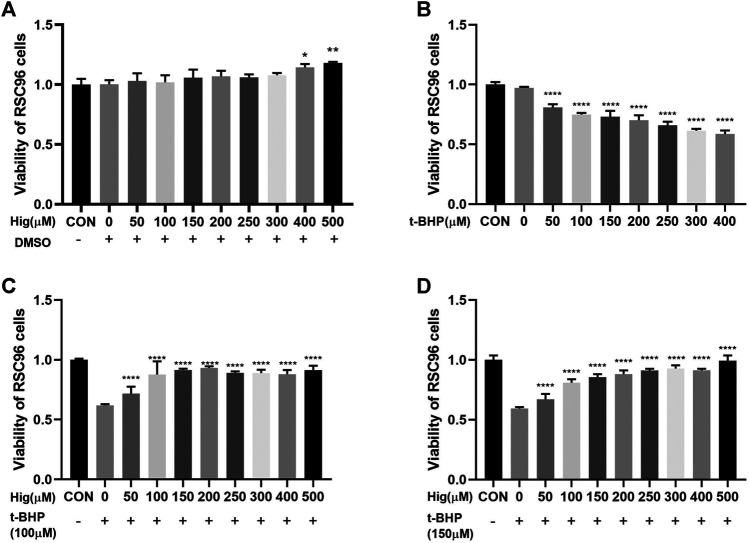
Cell viability of Hig and t-BHP treatment. **(A)** CCK-8 detected the cell viability of RSC96 treated with 50, 100, 150, 200, 250, 300, 400, and 500 µM Hig for 12 h. A DMSO control group was set up to exclude the potential cell cytotoxicity of DMSO. **(B)** CCK-8 detected the cell viability of RSC96 treated with 50, 100, 150, 200, 250, 300, and 400 µM t-BHP for 2 h. **(C)** RSC96 was pretreated with 50, 100, 150, 200, 250, 300, 400, and 500 µM Hig for 12 h and then, respectively, exposed to 100 µM t-BHP for 2 h. **(D)** RSC96 was pretreated with 50, 100, 150, 200, 250, 300, 400, and 500 µM Hig for 12 h and then, respectively, exposed to 150 µM t-BHP for 2 h. CCK-8 was used to assess cell viability. The results are presented as mean ± SEM, *n* = 3. *p < 0.05, **p < 0.01, and ****p < 0.0001 vs. the control group.

CCK-8 was used to evaluate cell viability with different concentration of Hig (50, 100, 150, 200, 250, 300, 400, and 500 µM). As shown in Figure 1A, Hig treated RSC96 at six concentrations for 12 h with no significant difference compared to the control group but 400 and 500 µM concentrations significantly enhanced cell viability. T-BHP decreased obviously the viability of RSC96 at 2 h with 50, 100, 150, 200, 250, 300, and 400 µM, which meant that different concentration even the lowest concentration (50 µM) would increase the risk of cell death (Figure 1B). Then, we assessed the underlying protective effect of Hig against t-BHP-induced cell cytotoxicity. The pretreated RSC96 at 50, 100, 150, 200, 250, 300, 400, and 500 µM concentration was exposed to t-BHP at the concentration of 100 and 150 µM for 2 h. Compared with the model group (t-BHP treatment), a dose-dependent manner of cell viability which significantly increased was observed in pretreated Hig at different concentration (Figures 1C,D). The final dosing concentration of t-BHP and Hig was determined to be 150 µM and 100/200/400 µM, respectively.

### Hig Attenuated Oxidative Stress Injury *In Vivo* and *In Vitro*

**FIGURE 2:**
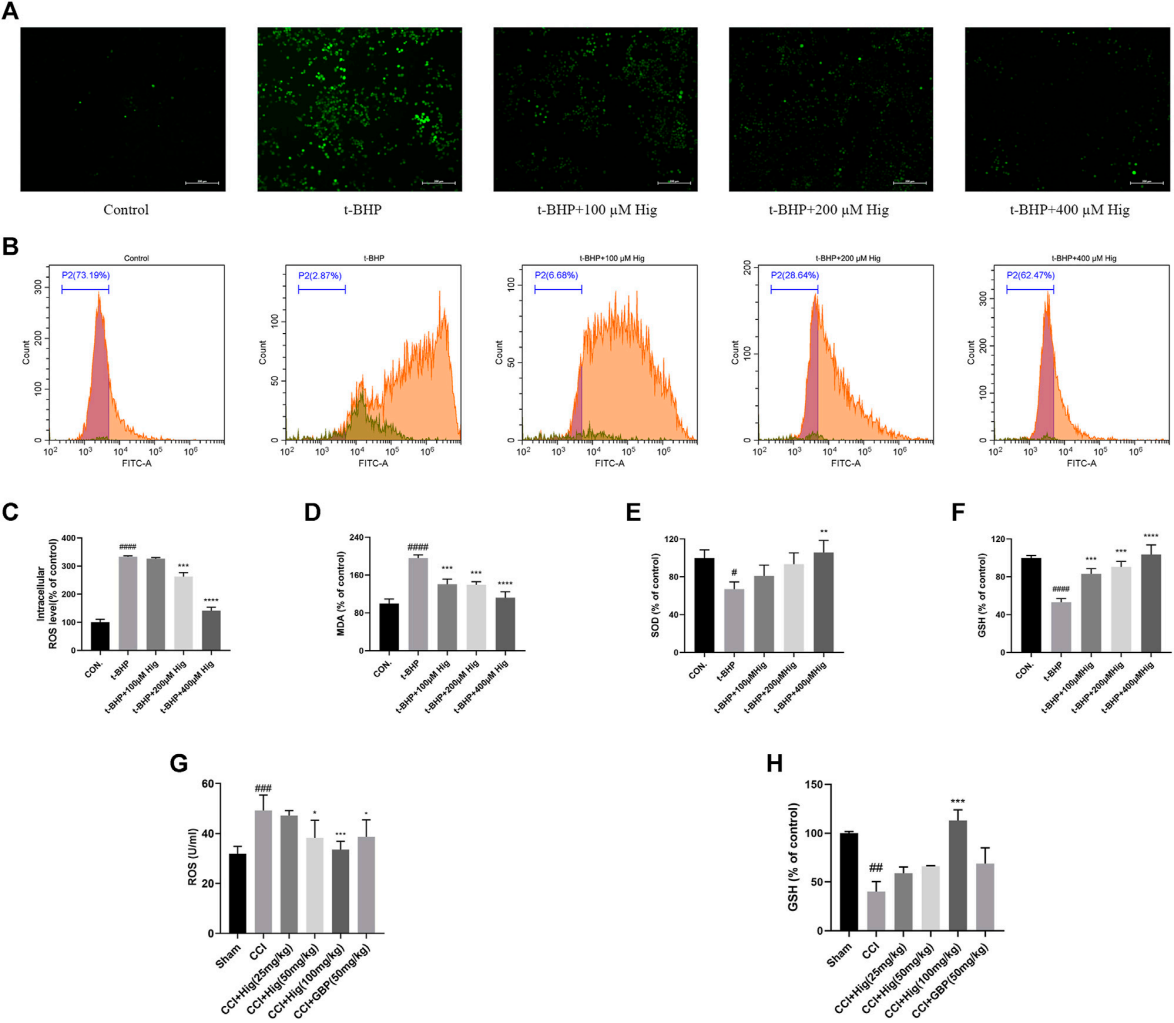
Hig prevented RSC96 and DRG neuron from oxidative stress damage. **(A)** RSC96 intercellular level of ROS stained by DCFH-DA dye was observed under the microscope. Scar bar = 200 µm. **(B)** RSC96 intercellular level of ROS stained by DCFH-DA dye was observed by flow cytometer. **(C)** The ratios of RSC96 intercellular ROS level compared with the control group were calculated. **(D)** Quantification of RSC96 intercellular MDA level in different groups. **(E)** Quantification of RSC96 intercellular SOD level in different groups. **(F)** Quantification of RSC96 intercellular GSH level in different groups. **(G)** The ROS level in rats’ DRG tissue was detected by ELISA kit. **(H)** Comparison of GSH level in rat DRG tissues among different groups. The results are presented as mean ± SEM, *n* = 3. #*p* < 0.05, ##*p* < 0.01, ###*p* < 0.001, and ####*p* < 0.0001 vs. the control group. *p < 0.05, **p < 0.01, ***p < 0.001, and ****p < 0.0001 vs. the model group.

To explore the antioxidant properties of Hig, we adopted the model of t-BHP-induced RSC96 oxidative stress *in vitro* and CCI-induced DRG neuron damage *in vivo*. From the results shown in Figures 2A–F, t-BHP treated alone for 2 h significantly increased intercellular levels of ROS and MDA but reduced SOD and GSH levels, which indicated that t-BHP imbalanced the redox system and caused the dysfunction of RSC96. However, pretreatment with Hig reversed these changes compared to the model group. Also, ROS and GSH were mainly evaluation indexes in DRG neuron. In Figures 2G,H, the level of ROS in DRG was upregulated by CCI surgery and the content of GSH was reduced. After 21 days of treatment with Hig, the upregulated ROS was significantly decreased by medium and high concentration (50/100 mg/kg) of Hig, and the downregulated GSH was obviously increased by a high concentration of Hig.

### Hig Decreased T-BHP-Induced RSC96 Apoptosis

**FIGURE 3:**
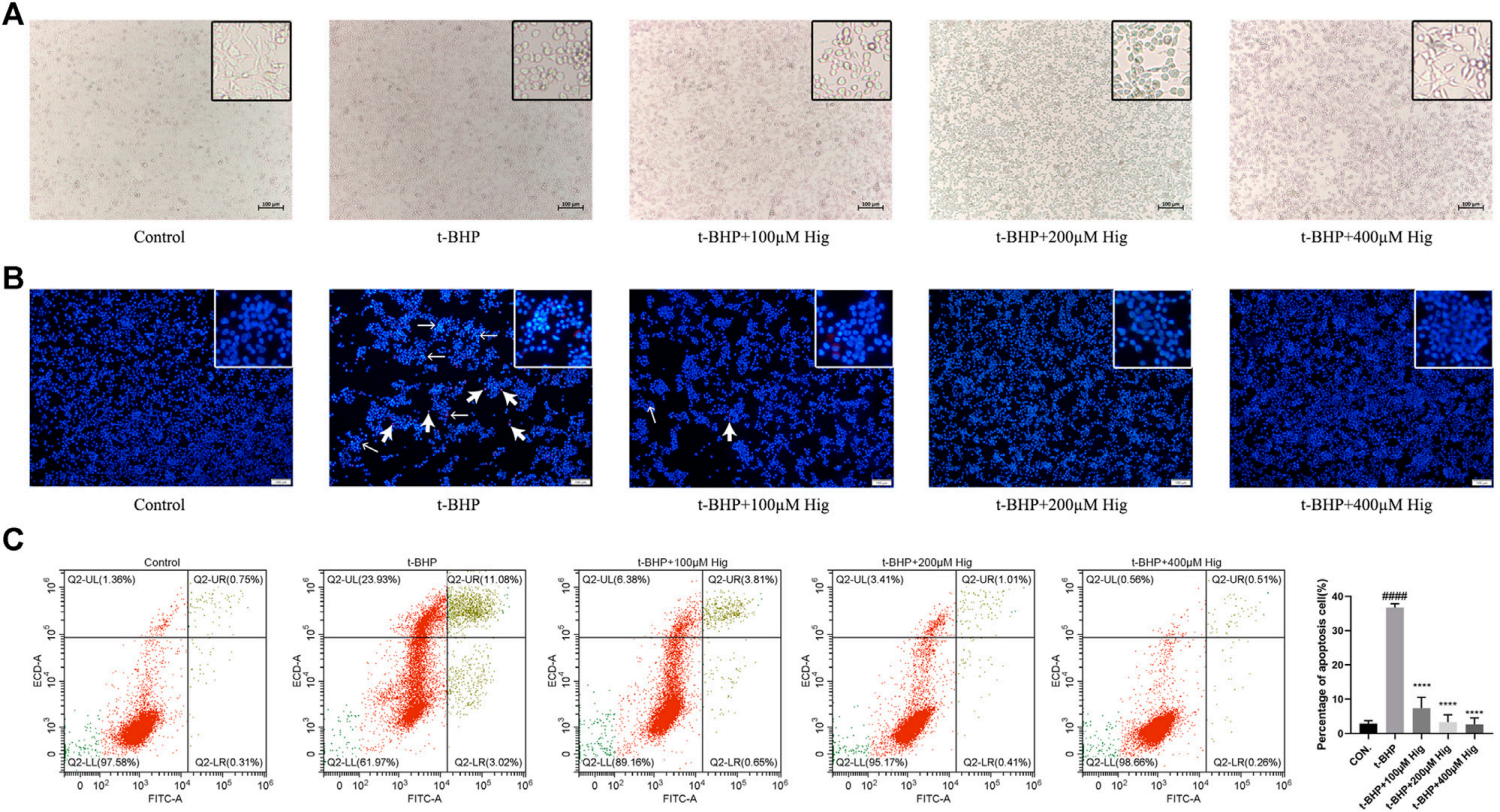
Hig protected RSC96 against t-BHP-induced cell apoptosis. **(A)** The typical morphological images in different groups. **(B)** The stained RSC96 by Hoechst 33258/PI dye was observed under the microscope. The nucleus of RSC96 marked with strong blue (the thin black arrows) and red (the thick black arrows) fluorescence represented cell apoptosis and necrosis, respectively. Scar bar = 100 µm. **(C)** Annexin V-FITC/PI apoptosis kit analyzed the cell apoptosis *via* flow cytometer and the percentage of RSC96 apoptosis was calculated from the addition of Q2-UL, Q2-UR, and Q2-LR. The results are presented as mean ± SEM, *n* = 3. ####*p* < 0.0001 vs. the control group. ****p < 0.0001 vs. the t-BHP group.

Morphologically, RSC96 in the control group appeared healthy with rich synapse. Apparent cell shrinkage was observed in RSC96 after 2 h treatment with t-BHP but those cells pretreated with Hig remained in relatively healthy appearance compared to the model group (Figure 3A). Hoechst 33258/PI staining and flow cytometry assay were applied to detect the apoptosis condition of RSC96 exposed to t-BHP. According to Figure 3B, most of the cell nuclei were densely stained with strong blue fluorescence and partly with red fluorescence, indicating that t-BHP could induce the apoptosis and necrosis rate of RSC96. Flow cytometry was usually used to detect the apoptosis ratio by using Annexin V-FITC/PI assay kit. In Figure 3C, the sum of quadrants Q2-UR and Q2-LR was representing the percentage of apoptotic cells, and the value of quadrant Q2-UL was representing the percentage of necrotic cells. Therefore, the same as the result of Hoechst 33258/PI staining, t-BHP treatment increased the apoptotic and necrotic rate of RSC96.

### Hig Inhibited the Expression Level of Inflammation-Related Mediators

**FIGURE 4:**
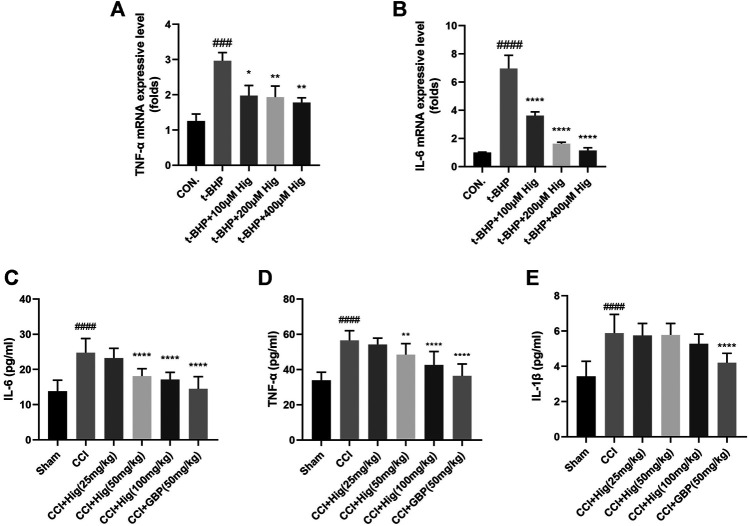
The anti-inflammatory function of Hig was evaluated by its effect on the expression of inflammatory factors. **(A, B)** The RSC96 intercellular mRNA expression levels of TNF-α and IL-6 were measured by RT-qPCR. **(C–E)** The expression levels of TNF-α, IL-6, and IL-1β in rats serum were detected by specific ELISA kits. The results are presented as mean ± SEM, *n* = 6. ###*p* < 0.001 and ####*p* < 0.0001 vs. the control group. *p < 0.05, **p < 0.01, ***p < 0.001, and ****p < 0.0001 vs. the model group.

It is known that overproduction of ROS during oxidative stress results in chronic inflammation (Willcox et al., 2004). We had checked whether Hig decreased the expression level of TNF-α, IL-6, and IL-1β after t-BHP treatment and CCI surgery. The results were shown in Figures 4A–E. Indeed, t-BHP treatment and CCI surgery upregulated the expression level of TNF-α, IL-6, and IL-1β (the expression level of IL-1β in cells was not shown in Figure 4). Pretreatment with Hig decreased the high expressive level of TNF-α and IL-6 but did not show the significant difference of reducing IL-1β expression level compared with the control group. The results proved that TNF-α and IL-6 were the potential inflammatory targets attenuated by Hig.

### Hig Alleviated Mechanical Allodynia and Heat Hyperalgesia in CCI Rats

**FIGURE 5:**
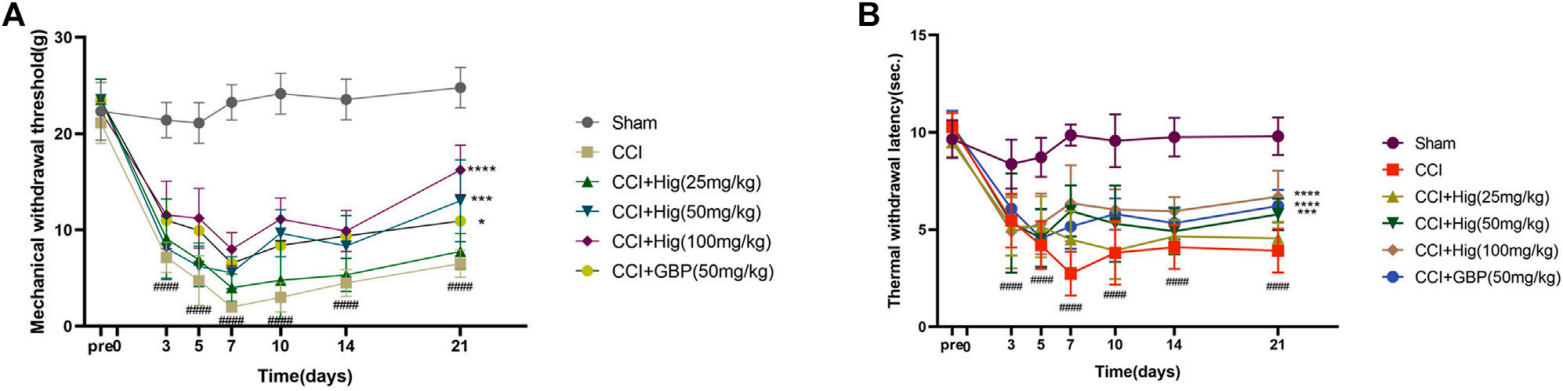
Hig relieved mechanical allodynia and heat hyperalgesia in CCI rats by raising the thresholds of MWT and TWI. **(A)** The value of MWT in each group was detected by the von Frey test on the day before the surgery and days 3, 5, 7, 10, 14, and 21 after surgery. **(B)** The value of TWI in each group was detected by the hot-plate test. The results are presented as mean ± SEM, *n* = 8. ####*p* < 0.0001 vs. the control group. *p < 0.05, ***p < 0.001, and ****p < 0.0001 vs. the CCI group.

CCI surgery led to obvious mechanical allodynia and heat hyperalgesia and they were detected by von Frey test and hot-plate test, respectively. As shown in Figures 5A,B, mechanical withdrawal threshold (MWT) and thermal withdrawal latency (TWI) both decreased significantly at day 3 after CCI surgery and reached the lowest value on day 7 after the operation. On day 7, obviously, a difference was observed between the high-concentration Hig and CCI groups. After 21 days of treatment, all groups but the low-concentration Hig group showed markedly pain relief effect by reducing mechanical allodynia and heat hyperalgesia in CCI rats.

### Hig Protected the Injured Sciatic Nerve in CCI Rats

**FIGURE 6:**
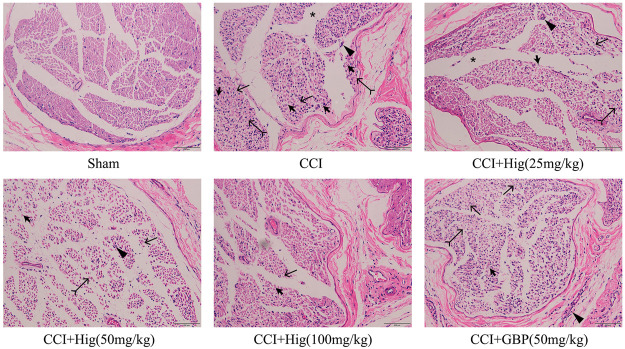
The typical pathological images of DRG tissue in CCI rats. Thick arrow, thin arrow, bifid arrow, triangle arrow, and star symbols illustrate Schwann cell (SC) nuclei, myelin vacuolation, changes of axoplasm, inflammatory cell infiltration, and wide separation between the nerve fibers, respectively. Scar bar = 200 µm.

As shown in Figure 6, the sham-operated group had no obvious pathological changes. The sciatic nerve tissue in the CCI group had shown the nerve fiber structural disorder accompanied by neuron loss, degeneration, and nuclear pyknosis. Myelin vacuolation, proliferation of SC, and inflammatory cell infiltration were also observed by a microscope. After Hig treatment, the above pathological changes were improved in a dose-dependent manner and the occurrence of neuron loss, degeneration, and nuclear pyknosis was inhibited. However, the GBP group, as the positive drug control group, only showed a few improvement effects on the above condition.

### Hig Showed the Protective Function in RSC96 and DRG Neuron by Regulating the Mitochondrial Apoptosis Pathway

**FIGURE 7:**
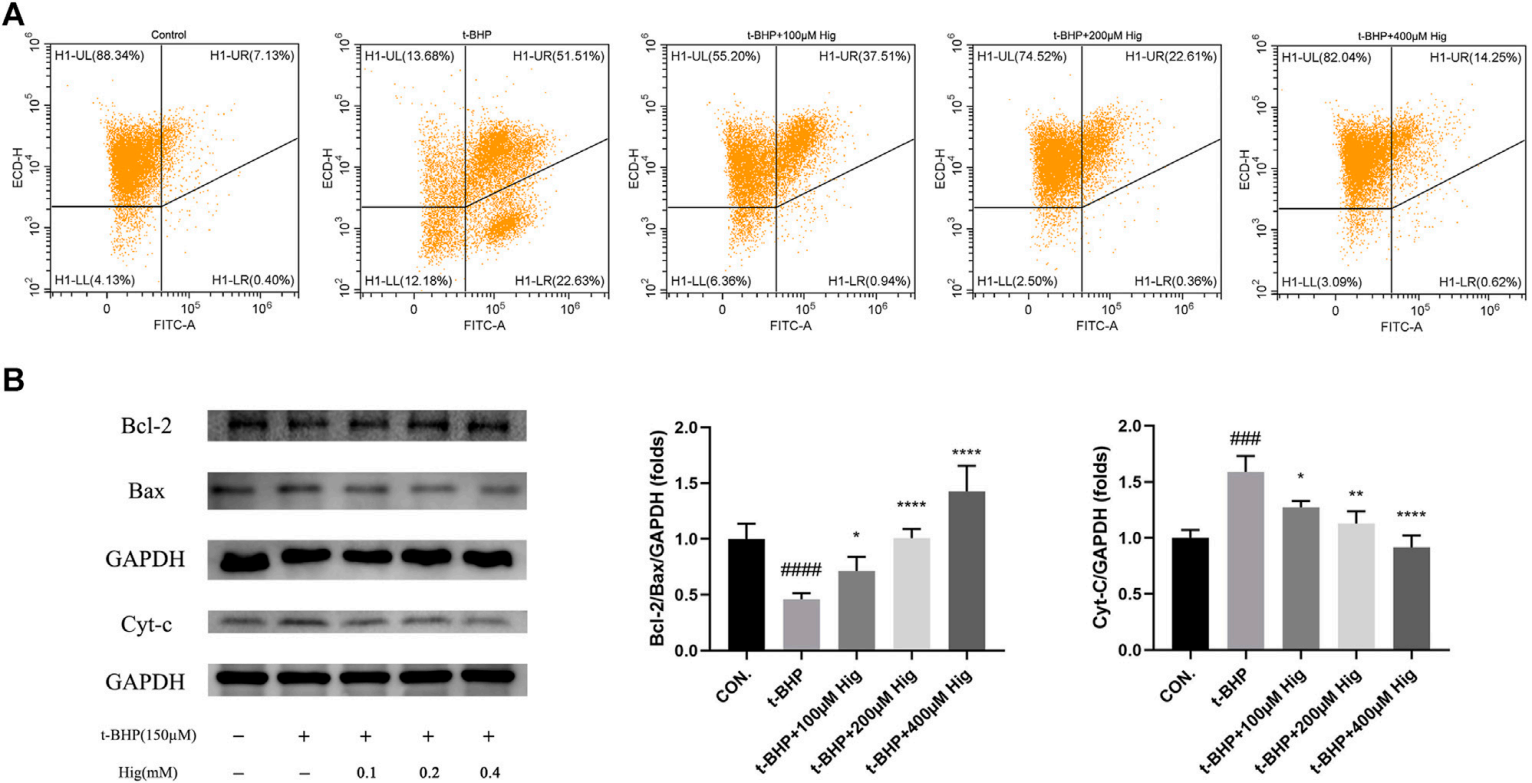
Hig protected RSC96 against oxidative stress-induced apoptosis by regulating mitochondrial pathway. **(A)** △Ψm was detected by JC-1 probe using flow cytometer. The sum of quadrants H1-LL and H1-LR represented the depolarization extent of △Ψm. **(B)** Protein bands were detected by WB analysis. The ratios of Bcl-2/Bax protein level and the expression level of cyt-c protein were quantified. GAPDH served as an internal control. The results are presented as mean ± SEM, *n* = 3. ###*p* < 0.001 and ####*p* < 0.0001 vs. the control group. *p < 0.05, **p < 0.01, and ****p < 0.0001 vs. the t-BHP group.

**FIGURE 8:**
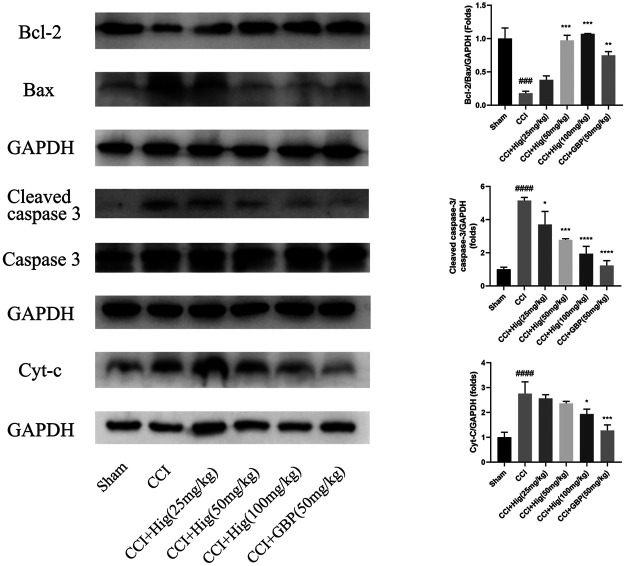
Hig protected DRG neuron against CCI-induced oxidative stress and apoptosis by regulating mitochondrial pathway. WB analysis was used to quantify the ratio of Bcl-2/Bax and cleaved caspase 3/caspase 3 protein level and the expression level of cyt-c protein. GAPDH was considered as an internal control. The results are presented as mean ± SEM, *n* = 3. ###*p* < 0.001 and ####*p* < 0.0001 vs. the control group. *p < 0.05, **p < 0.01, ***p < 0.001, and ****p < 0.0001 vs. the t-BHP group.

*In vitro* JC-1 probe was used to detect MMP (△Ψm), which was generally applied to assess early cell apoptosis. As shown in Figure 7A, compared with the control group, △Ψm depolarized after t-BHP stimulation and pretreatment with Hig reversed the depolarization of △Ψm and inhibited early cell apoptosis. WB analysis was performed to analyze the expression level of Bcl-2, Bax, and cytochrome-c (cyt-c) proteins in RSC96 and DRG tissues (Figures 7B, 8). The results showed that both t-BHP treatment and CCI operation reduced the expression level of the antiapoptotic protein Bcl-2 in RSC96 and DRG neurons and increased the proapoptotic proteins Bax and cyt-c. Pretreatment with Hig increased the ratio of bcl-2/bax and downregulated the expression level of cyt-c, indicating further neuroprotection of Hig on RSC96 and DRG neurons. In addition, Hig also decreased the expression level of cleaved caspase 3/caspase 3 in DRG neurons.

### Hig Regulated Nox2/ROS/TRP/P38 MAPK/NF-ĸB Signaling Pathway for Its Therapeutic Effect on Pain Relief, Antioxidation, Anti-Inflammatory

**FIGURE 9:**
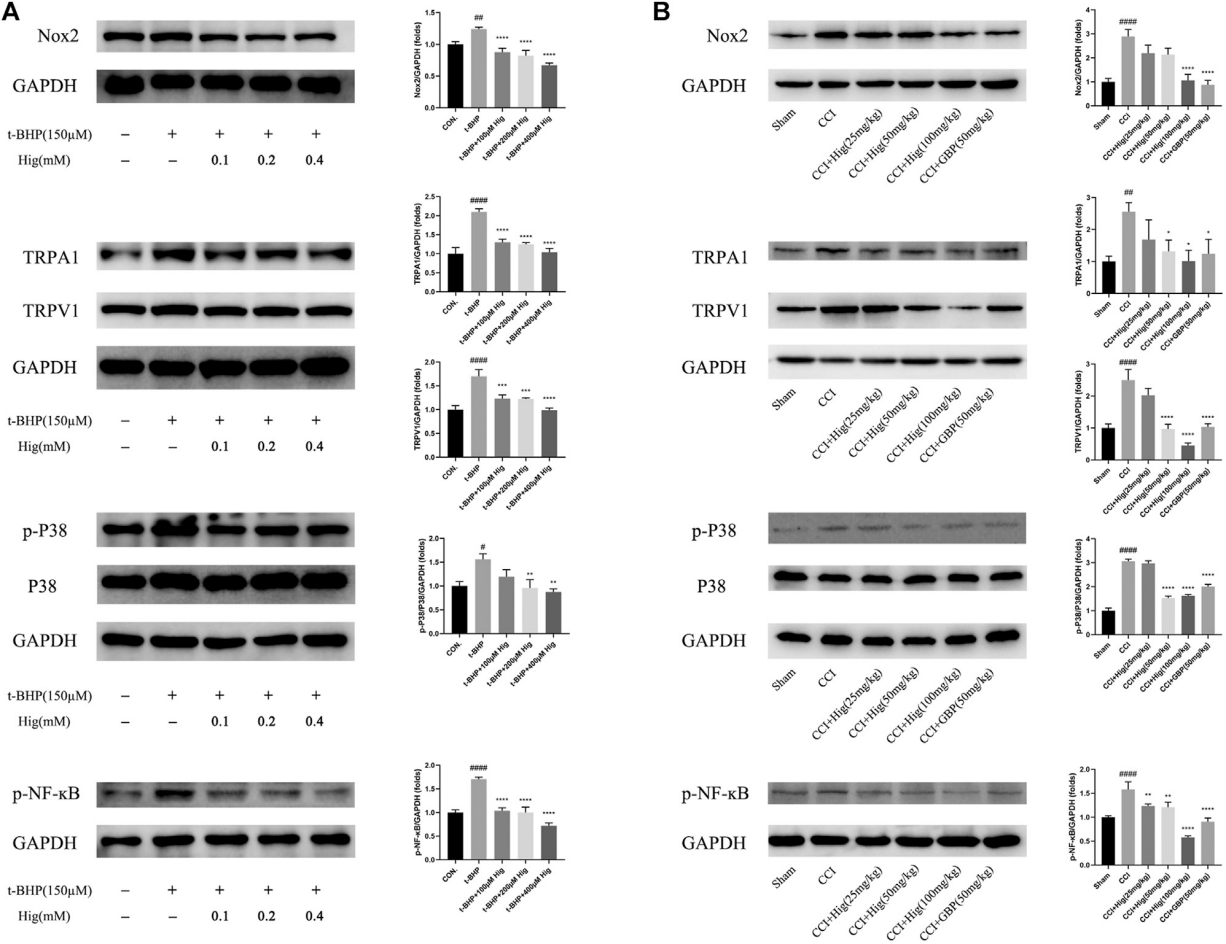
Hig regulated the proteins expressed in Nox2/ROS/TRP/P38 MAPK/NF-ĸB signaling pathway. **(A)** The control and t-BHP-exposed RSC96 were collected and analyzed by WB for expression level of Nox2, TRPA1, TRPV1, p38 MAPK, p-p38 MAPK, and p-NF-ĸB. **(B)** The DRG tissues from sham-operated and CCI surgery were collected and analyzed by WB for expression level of Nox2, TRPA1, TRPV1, p38 MAPK, p-p38 MAPK, and p-NF-ĸB. GAPGH served as internal control. The results are presented as mean ± SEM, *n* = 3. #*p* < 0.05, ##*p* < 0.01, and ####*p* < 0.0001 vs. the control group. *p < 0.05, **p < 0.01, ***p < 0.001, and ****p < 0.0001 vs. the model group.

To explore the underlying molecular mechanism of Hig on treating t-BHP-exposed RSC96 and CCI model rats, we detected the expression level of Nox2, TRPA1, TRPV1, p38 MAPK, p-p38 MAPK, and p-NF-ĸB using WB analysis. The results were shown in Figures 9A,B. After t-BHP treatment alone, an obviously upregulating level of Nox2, TRPA1, TRPV1, p-p38 MAPK, and p-NF-ĸB was observed from the protein bands. Pretreated Hig inhibited the rising trend of the above proteins. The same consequence was also observed *in vivo*. The expression levels of Nox2, TRPA1, TRPV1, p-p38 MAPK, and p-NF-ĸB proteins were increased in CCI rats while there was a declining tendency of these proteins after 21 days of treatment with Hig. The results showed that Hig played an important role in suppressing the protein expression level of Nox2, TRPA1, TRPV1, p-p38 MAPK, and p-NF-ĸB.

**FIGURE 10:**
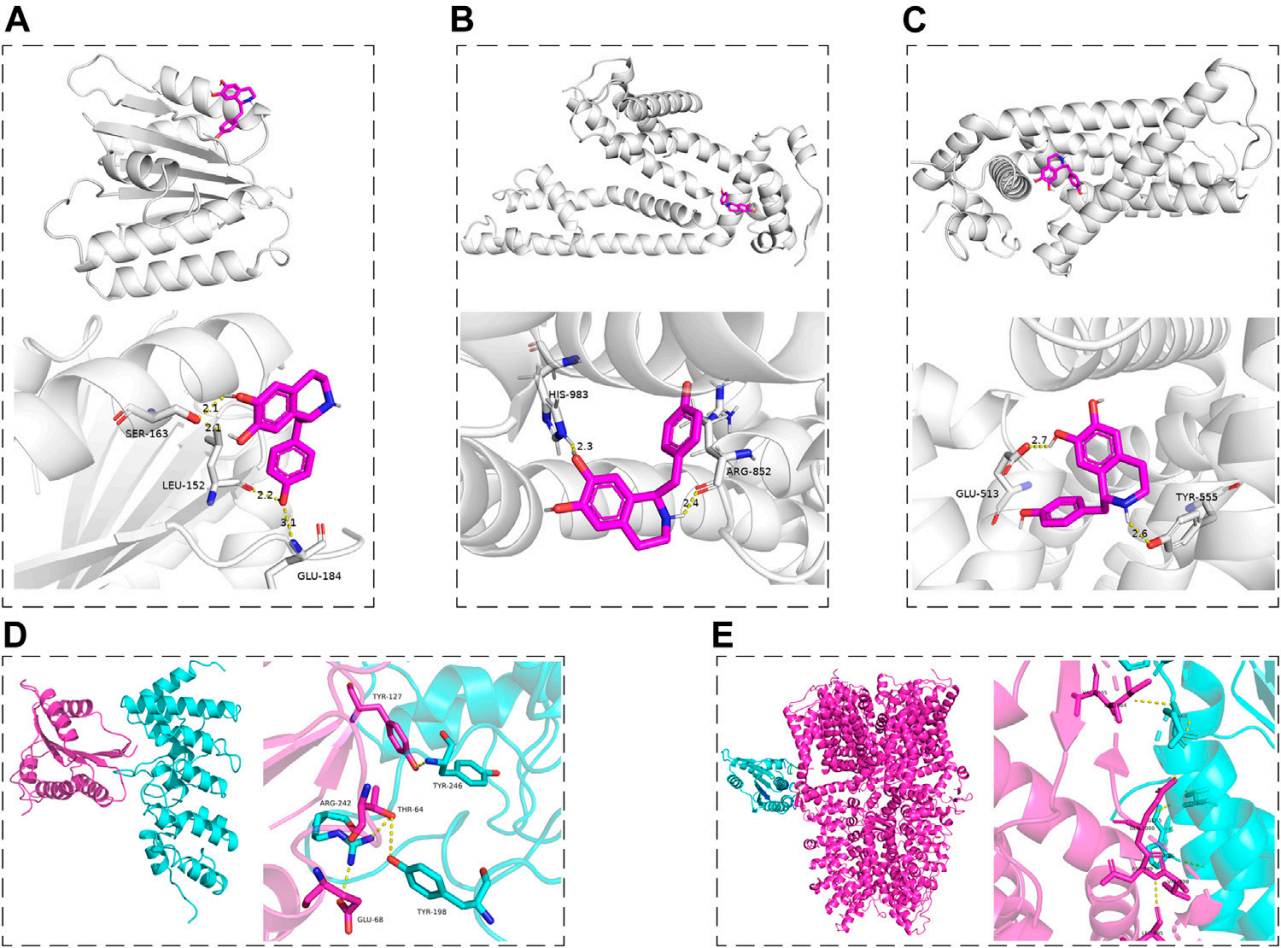
The molecular docking maps. **(A–C)** The three-dimensional structure of Hig combined with Nox2, TRPA1, and TRPV1, respectively. **(D, E)** The schematic diagram of the optimal complex in Nox2-TRPV1 and Nox2-TRPA1 protein docking. The dashed line indicated the hydrogen bond formed between Hig and amino acid residues and the length of the hydrogen bond.

According to the results of this work and literature reported (Puntambekar et al., 2005; Marone et al., 2018), we assumed that some connection would exist between Nox2 and TRPV1, Nox2, and TRPA1. The molecular docking was used for preliminary verification and model creation to provide further proof for subsequent experiments. The molecular docking maps were shown in Figure 10 and the binding sites of Nox2, TRPV1, and TRPA1 protein were listed in Tables 2, 3. The scores for the Hig-Nox2, Hig-TRPV1, and Hig-TRPA1 molecule-protein docking were −6.0 kcal mol^−1^, −8.0 kcal mol^−1^, and −8.8 kcal mol^−1^, suggesting that Hig was tightly bound to Nox2, TRPV1, and TRPA1. Hig formed a hydrogen bond with a bond length of 2.1, 2.2, and 3.1 Å in Nox2 protein amino acids SER163, LEU152, and GLU184, respectively (Figure 10A), a hydrogen bond with a bond length of 2.3 and 2.4 Å in TRPA1 protein amino acids HIS983 and ARG852, respectively (Figure 10B), and a hydrogen bond with a bond length of 2.6 and 2.7 Å in TRPV1 protein amino acids TYR555 and GLU513, respectively (Figure 10C). The protein-protein docking was performed to detect the probably connective binding sites between Nox2 and TRPV1, Nox2 and TRPA1. The amino acids TYR246, TYR198, and ARG242 in TRPV1 were combined with amino acids TYR127, THR64, THR64, and GLU68 in Nox2 to form stable hydrogen bonds (Figure 10D). The optimal score in the complex was 1617.495. As for Nox2-TRPA1, the amino acids VAL1005, ILE1004, GLN1000, ASP999, VAL998, and LEU995 in TRPA1 were combined with amino acids GLN68, GLY-5, and PRO6 in Nox2 to form stable hydrogen bonds (Figure 10E). The optimal score in the complex was 2055.496.

**TABLE 2:** The binding sites of Nox2 and TRPV1 protein.

TRPV1	Nox2
TYR246	TYR127
TYR198	THR64
ARG242	THR64 and GLU68

**TABLE 3:** The binding sites of Nox2 and TRPA1 protein.

TRPA1	Nox2
VAL1005 and ILE1004	GLN68
GLN1000	GLY-5
ASP999, VAL998, and LEU995	PRO6

## Discussion

NP seriously affects the quality of life of patients. It is found that the incidence of NP rises to 3–17% in the recent statistical analysis of epidemiology (van Hecke et al., 2014). Most of the therapies have little benefit to patients and are always accompanied by side effects. Therefore, finding an effective drug for treating NP is the key breakthrough point. However, the pathogenesis of NP is very complicated and there is still no exact statement to reveal the specific mechanism. The development and maintenance of NP not only are related to central and peripheral sensitization but also involve the accumulated products of oxidative stress and inflammatory response, as well as the highly interweaving signaling pathway among neurons, Schwann cells (SC), and immune cells. More and more studies support the view that oxidizing substances (including superoxide and hydrogen peroxide) drive the generation of NP and it is believed that inhibiting the production of oxidizing substances is a potential treatment to alleviate NP (Grace et al., 2016). Many studies have verified that Hig possesses a strong antioxidant effect through *in vivo* and *in vitro* experiments (Guan et al., 2019; Wang et al., 2019; Wen et al., 2019; Guler et al., 2020). In the current study, Hig protected RSC96 against t-BHP-induced oxidative stress and showed the antinociceptive and neuroprotective activities in the rat with CCI.

SC, the most abundant peripheral glial cells, respond to peripheral nerve injury by the way of changing phenotype and releasing of inflammatory mediators (TNF-α, IL-6, and IL-1β), which leads to the exacerbation of NP (Shamash et al., 2002; Kobayashi et al., 2008). Targeted SC also is regarded as a positive therapy for alleviating NP. Thus, RSC96 was chosen to carry on *in vitro* experiments to evaluate drug efficacy. In the study, we found that the exogenous ROS donor (t-BHP) imbalanced the redox system, which is manifested in the excessive release of ROS and MDA, the reduction of SOD and GSH, even the decrease of MMP, and the increase of cell apoptosis and necrosis. However, these changes were reversed after Hig medication.

CCI, the typical model-induced NP, was adopted to further evaluate the antioxidant, analgesic, and neuroprotective properties of Hig. CCI caused inflammation and hyperalgesia in rats, which was consistent with our previous research results (Zhang et al., 2020). In addition, a high level of ROS and low expression of GSH in DRG neurons were observed, indicating that CCI surgery triggered oxidative stress injury in local tissue. Histopathological evaluation of the H & E staining of sciatic nerve sections was applied to illustrate the neuroprotective effect of Hig. The results showed that Hig improved the pathological changes of the sciatic nerve in CCI rats, alleviated thermal hyperalgesia and mechanical allodynia caused by CCI, and attenuated inflammation and oxidative stress in rats.

Mammalian nerves are more vulnerable at an oxidative stress condition for the abundant phospholipids and mitochondria-rich axoplasm (Areti et al., 2014). ROS is a product of the normal metabolism of oxygen in human body. ROS acts as a functional messenger molecule to maintain the normal operation of cell activities (Kallenborn-Gerhardt et al., 2013). Under pathological conditions, a large amount of ROS will destroy cell signal transduction pathways, oxidize proteins and lipid cells, fragment DNA, cause tissue damage, and induce irreversible effects. Nicotinamide adenine dinucleotide phosphate oxidase (Nox) is the place that produces ROS and the generation of ROS seems to be its main function. Nox2 is one of the main phenotypes of the enzyme. It is found that NOX2-ROS could cause excessive excitement of DRG neurons in the SNI animal model and promote the plasma membrane translocation of PKCε to induced NP. The use of the specific NOX2 inhibitor gp91-tat could alleviate the above performance (Xu et al., 2021). To well understand the possible potential antioxidant mechanism of Hig in oxidative damage, the experiment also evaluated the protein expression level of Nox2 and discovered that the expression of Nox2 was upregulated in the t-BHP-stimulated RSC96 model. The same result also appeared in DRG neurons. Hig reversed the situation and reduced the generation of ROS, which may be related to its inhibition of Nox2 activity.

Mitochondria are the order place that generates free radicals. Endogenous ROS accumulates in normal respiration or certain dysfunctions. The high potential of △Ψm triggers the opening of mitochondrial pores to discharge ROS for preventing excessive accumulation of ROS in mitochondria (Zorov et al., 2014). However, the emission of ROS in mitochondria is irreversible in certain pathological processes. At this time, mitochondria will lead to a great quantity of release of ROS, reducing cell apoptosis or autophagy. Once the apoptosis program is initiated, cytochrome-c will translocate from mitochondria to cytoplasm, driving the features of subsequent apoptosis, for instance, the activation of caspase 3 and the overexpression level of proapoptotic protein. Our study results suggested Hig reduced the apoptosis rate of RSC96 and DRG neurons by acting on the mitochondrial apoptosis pathway. It meant that Hig blocked the release of cyt-c and improved the expression of antiapoptotic protein Bcl-2. There was a research that reported that cell necrosis will be induced by significantly increasing levels of intracellular ROS levels (Yeh et al., 2020). Interestingly, a significant increase in the necrosis rate of RSC96 was observed after t-BHP treatment for 2 h and pretreatment of Hig protected RSC96 against cell necrosis. Maybe the protective effect is related to its excellent antinecrotic activity but further exploration in subsequent studies should be needed.

The transient receptor potential (TRP) ion channel is one of the abundant ion channels in DRGs. Because of its strong selectivity and wide participation in the process of pain production, it has become a popular target for NP drug screening in recent years. The TRP family represented by TRPV1 and TRPA1 is a nonselective cation channel that could be modulated by harmful stimuli such as ROS (Nishio et al., 2013). TRPV1 is the first TRP ion channel to be discovered. It is widely expressed in tissues and organs (bladder, lungs, blood vessels, and so on) and considered to be closely related to the generation of inflammatory hyperalgesia and thermal hyperalgesia (Sanchez et al., 2001). After peripheral nerve injury, the release of inflammatory substances lowers the thermal and mechanical thresholds of sensory neurons, which will sensitize the exposed neurons (Julius, 2013). TRPA1 is another TRP family member playing an important role in mediating mechanical hyperalgesia and cold hyperalgesia (Obata et al., 2005). In DRG neurons, TRPA1 was observed upon coexpression with TRPV1, both of which induced the activation of sensory nerves and evoked pain and neuroinflammation (Fernandes et al., 2012). All these could result in the persistent pain. TRPA1 and TRPV1 are activated by Nox-dependent ROS release (Ibi et al., 2008; De Logu et al., 2017). Our previous work also revealed an upregulated tendency of TRPA1 and TRPV1 after the stimulus of H_2_O_2_ (another ROS donor) (Zhang et al., 2021). In the study, we mainly detected the expression of TRPA1 and TRPV1 in DRG and RSC96. The results showed a high expression level of TRPA1 and TRPV1 in the cells and animal model group. Hig pretreatment reversed these changes, which may be related to its targeting of these ion channels.

The chronic overproduction of ROS is an important substance in the progression of inflammatory diseases (Mittal et al., 2014). It causes the production and secretion of inflammatory mediators (TNF-α; interleukins), which are activating the proinflammatory signal transduction pathway (NF-ĸB signaling pathway) and promoting the expression of other proinflammatory genes (Lawrence, 2009). p38 MAPK also is the potential downstream target of ROS (Kallenborn-Gerhardt et al., 2013). Many growth factors produced by inflammation are transported to the nociceptors in DRG through the intracellular signaling pathway p38 MAPK to increase the expression of TRP ion channels and then they are transported to peripheral nerves resulting in peripheral sensitization (Patapoutian et al., 2009). Our experimental results indicated that excessive ROS production could lead to inflammatory response accompanied by an increased tendency of phosphorylation of P38 MAPK and NF-ĸB in RSC96 and DRG neurons. Hig restrained the phosphorylation level of NF-ĸB and inhibited the release of inflammatory mediators to show its anti-inflammatory activity. However, Hig only showed an inhibitory effect on the two inflammatory factors, TNF-a and IL-6, but no statistical significance in the inhibition of IL-1β.

Hig could be extracted from a variety of botanicals, such as AsariRadix Et Rhizoma, Linderae Radix, and *Aconitum carmichaeli* Debx. As a tetrahydroisoquinolinic (TIQ) derivative, it is provided with neuroprotective property for its unique structure (Peana et al., 2019). Previous studies reported that TIQ derivatives are the ligands of Nox2, TRPV1, and TRPA1 (O’Dell et al., 2007; Cifuentes-Pagano et al., 2013; Kistner et al., 2016). In particular, the core pharmacophore of the first TRPV1 antagonist, capsazepine 3, is the tetrahydroisoquinoline moiety (O’Dell et al., 2007). Our study demonstrated that Hig had good inhibitory effects on the above proteins. It is reported that endogenous ligands and TRPV1 antagonists act on the vanilloid-pocket of TRPV1 (transmembrane domain structure formed by Ser505–Thr550) (Szolcsányi and Sándor, 2012) and the molecules bound to these pocket areas appeared to inhibit the activity of TRPV1 for reducing pain condition (Zheng et al., 2021). The binding pocket near amino acid residues Trp711, Ile858, Val861, Val967, Met978, and Leu982 could be linked with the TRPA1 antagonist (Terrett et al., 2021), which means that targeting the pocket also showed a pain relief effect (Melo et al., 2017; Lee et al., 2020). However, there is little discussion about the active pocket of Nox2 protein at present, so we predicted the possible binding sites of Nox2 by using the DeepSite of PlayMolecule website. The molecular docking was used to verify the hypothesis that Hig may act as a blocker of Nox2, TRPV1, and TRPA1 proteins. The results showed that Hig interacted with the protein through the formation of bond ranges and binding energy in these specific structural regions, indicating that it had the potential to become the antagonist of Nox2, TRPA1, and TRPV1, but further experimental verification is needed. Although Hig formed strong hydrogen bonds with these proteins, further experimental verification is needed. Based on our existing research results, Hig inhibited the activity of Nox2 while the content of TRPA1 and TRPV1 were also been decreased. We assume that there may be some interaction between Nox2 -TRPA1 and/or Nox2-TRPV1. Recent studies reported that Nox2 and TRPA1 were tightly located in the cell bodies of trigeminal ganglion neurons by using proximity ligation assay and speculated that their interaction could be the underlying cause for the effective release of ROS (Marone et al., 2018). The molecular docking was carried out to analyze Nox2-TRPA1 and Nox2-TRPV1. Both of them form stable complexes, which provides novel ideas for our subsequent research on Nox2-TRP. Besides, we used GBP, the first-line treatment for NP, as the positive control group in CCI rats. GBP functions as pain relief by targeting the α2δ-1 subunit of voltage-dependent calcium channels as well as TRPs, inflammatory cytokines, and N-methyl-D-aspartic acid (NMDA) receptors (Kukkar et al., 2013; Alles and Smith, 2018). The fact is the therapeutic effect of Hig showed similar efficacy profiles as GBP in reducing the expression level of TRPA1/TRPV1/TNF-α/IL-6. But other possible mechanisms of Hig on NP deserve our attention, for example, the potential NMDA receptors antagonist or/and α2δ-1 ligand.

In conclusion, it was the first time to verify the neuroprotective effect of Hig *via in vivo* and *in vitro* experiments. Hig acts as a potential candidate drug for NP treatment and probably reacted its function by inhibiting Nox2/ROS/TRP/p38 MAPK/NF-ĸB signaling pathway. However, further experiments are required to expound the complete molecular mechanism.

## Data Availability

The original contributions presented in the study are included in the article/supplementary material; further inquiries can be directed to the corresponding author.
